# 1*H*-Indole-3-carbaldehyde thio­semi­carbazone

**DOI:** 10.1107/S1600536808011082

**Published:** 2008-04-26

**Authors:** Razali M. Rizal, Hapipah M. Ali, Seik Weng Ng

**Affiliations:** a Department of Chemistry, University of Malaya, 50603 Kuala Lumpur, Malaysia

## Abstract

The mol­ecules of the title compound, C_10_H_10_N_4_S, are linked by *N*—H_indole_⋯S hydrogen bonds to form a linear hydrogen-bonded chain. There are two independent mol­ecules in the asymmetric unit.

## Related literature

For the synthesis and bateriostatic activity of indole-3-carbaldehyde semithio­carbazone, see: Doyle *et al.* (1956 ▶); Fujikawa *et al.* (1966 ▶); Libermann *et al.* (1953 ▶); Weller *et al.* (1954 ▶). For metal complexes of the compound, see: Bhardwaj & Singh (1994 ▶); Dalvi *et al.* (2004 ▶); Garg & Tandon (1988 ▶); Kanoongo *et al.* (1988 ▶, 1990 ▶); Kiran *et al.* (1986 ▶); Kumari *et al.* (1992*a* ▶,*b* ▶; 1993*a* ▶,*b* ▶); Rodriguez-Argueelles *et al.* (2005 ▶); Saxena & Singh (1994 ▶); Saxena *et al.* (1993 ▶, 1994 ▶); Singh & Singh (1990 ▶); Singh *et al.* (1987 ▶, 1988 ▶); Varshney & Tandon (1989 ▶); Varshney *et al.* (1989 ▶, 1996 ▶).
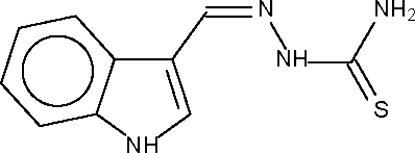


## Experimental

### 

#### Crystal data

C_10_H_10_N_4_S
*M* *_r_* = 218.28
Triclinic, 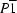

*a* = 7.1893 (1) Å
*b* = 11.1654 (2) Å
*c* = 13.5373 (3) Å
α = 68.887 (1)°
β = 85.048 (1)°
γ = 82.467 (1)°
*V* = 1004.07 (3) Å^3^
*Z* = 4
Mo *K*α radiation
μ = 0.29 mm^−1^
*T* = 123 (2) K
0.44 × 0.24 × 0.04 mm

#### Data collection

Bruker SMART APEX diffractometer
Absorption correction: multi-scan (*SADABS*; Sheldrick, 1996 ▶) *T*_min_ = 0.857, *T*_max_ = 0.988
9295 measured reflections
4527 independent reflections
3142 reflections with *I* > 2σ(*I*)
*R*_int_ = 0.036

#### Refinement

*R*[*F* ^2^ > 2σ(*F* ^2^)] = 0.044
*wR*(*F* ^2^) = 0.161
*S* = 1.10
4527 reflections
303 parameters
8 restraints
H atoms treated by a mixture of independent and constrained refinement
Δρ_max_ = 0.45 e Å^−3^
Δρ_min_ = −0.45 e Å^−3^

### 

Data collection: *APEX2* (Bruker, 2005 ▶); cell refinement: *SAINT* (Bruker, 2005 ▶); data reduction: *SAINT*; program(s) used to solve structure: *SHELXS97* (Sheldrick, 2008 ▶); program(s) used to refine structure: *SHELXL97* (Sheldrick, 2008 ▶); molecular graphics: *X-SEED* (Barbour, 2001 ▶); software used to prepare material for publication: *publCIF* (Westrip, 2008 ▶).

## Supplementary Material

Crystal structure: contains datablocks global, I. DOI: 10.1107/S1600536808011082/rz2196sup1.cif

Structure factors: contains datablocks I. DOI: 10.1107/S1600536808011082/rz2196Isup2.hkl

Additional supplementary materials:  crystallographic information; 3D view; checkCIF report

## Figures and Tables

**Table 1:** Hydrogen-bond geometry (Å, °)

*D*—H⋯*A*	*D*—H	H⋯*A*	*D*⋯*A*	*D*—H⋯*A*
N4—H4n⋯S1^i^	0.89 (1)	2.56 (2)	3.383 (3)	156 (3)
N8—H8n⋯S2^i^	0.88 (3)	2.49 (2)	3.325 (2)	157 (3)

Symmetry code: (i) 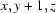.

**Fig. 1.**
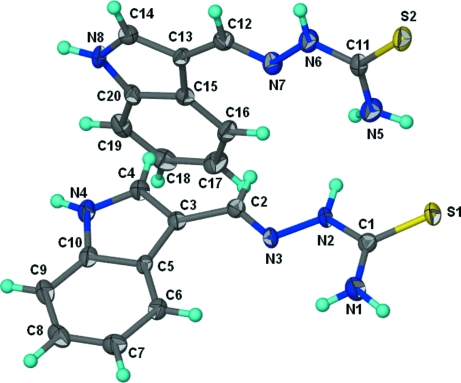
Thermal ellipsoid plot of the two independent molecules of the title compound. Displacement ellipsoids are drawn at the 70% probability level, and H atoms are shown as spheres of arbitrary radii.

**Fig. 2.**
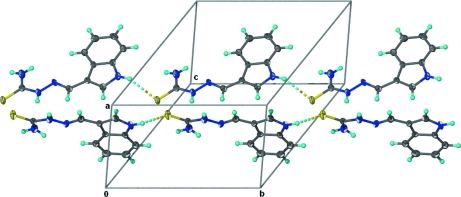
Chain structure of the title compound. Intermolecular H bonds are shown as dashed lines.

## supplementary crystallographic information

### Comment

The bacteriostatic activity of indole-3-carboxaldehyde thiosemicarbazone is known for a long time (Doyle *et al.*, 1956; Fujikawa *et al.*, 1966; Libermann *et al.*, 1953; Weller *et al.*, 1954). The compound yields complexes with main group as well as transition metal ions.

### Experimental

Thiosemicarbazide (0.3 g, 3.3 mmol) and indole-3-carboxaldehyde (0.5 g, 3.3 mmol) were refluxed in ethanol (50 ml) for 2 h. The solvent was removed to give the product Schiff base, and crystals were obtained upon recrystallization from ethanol.

### Refinement

The carbon-bound H atoms were placed at calculated positions (C–H 0.95 Å), and were included in the refinement in the riding model approximation with *U*(H) set to 1.2*U*_eq_(C). The amino H atoms were located in a difference Fouier map, and were refined with a distance restraint of N–H 0.88±0.01 Å.

### Crystal data

**Table:**

C_10_H_10_N_4_S	*Z* = 4
*M**_r_* = 218.28	*F*_000_ = 456
Triclinic, *P*1	*D*_x_ = 1.444 Mg m^−^^3^
Hall symbol: -P 1	Mo *K*α radiation λ = 0.71073 Å
*a* = 7.1893 (1) Å	Cell parameters from 4065 reflections
*b* = 11.1654 (2) Å	θ = 3.6–30.8º
*c* = 13.5373 (3) Å	µ = 0.29 mm^−^^1^
α = 68.887 (1)º	*T* = 123 (2) K
β = 85.048 (1)º	Wedge, colorless
γ = 82.467 (1)º	0.44 × 0.24 × 0.04 mm
*V* = 1004.07 (3) Å^3^	

### Data collection

**Table:**

Bruker APEXII diffractometer	4527 independent reflections
Radiation source: medium-focus sealed tube	3142 reflections with *I* > 2σ(*I*)
Monochromator: graphite	*R*_int_ = 0.036
*T* = 123(2) K	θ_max_ = 27.5º
φ and ω scans	θ_min_ = 1.6º
Absorption correction: multi-scan(SADABS; Sheldrick, 1996)	*h* = −8→9
*T*_min_ = 0.857, *T*_max_ = 0.988	*k* = −14→14
9295 measured reflections	*l* = −17→17

### Refinement

**Table:**

Refinement on *F*^2^	Secondary atom site location: difference Fourier map
Least-squares matrix: full	Hydrogen site location: inferred from neighbouring sites
*R*[*F*^2^ > 2σ(*F*^2^)] = 0.044	H atoms treated by a mixture of independent and constrained refinement
*wR*(*F*^2^) = 0.161	*w* = 1/[σ^2^(*F*_o_^2^) + (0.0864*P*)^2^ + 0.0582*P*] where *P* = (*F*_o_^2^ + 2*F*_c_^2^)/3
*S* = 1.10	(Δ/σ)_max_ = 0.001
4527 reflections	Δρ_max_ = 0.45 e Å^−^^3^
303 parameters	Δρ_min_ = −0.45 e Å^−^^3^
8 restraints	Extinction correction: none
Primary atom site location: structure-invariant direct methods	

### Special details

**Table:**

Experimental. A medium-focus collimator of 0.8 mm diameter was used on the diffractometer to measure the somewhat large crystal.

### Fractional atomic coordinates and isotropic or equivalent isotropic displacement parameters (Å^2^)

**Table:**

	*x*	*y*	*z*	*U*_iso_*/*U*_eq_	
S1	0.61344 (11)	0.11515 (7)	0.35357 (6)	0.0218 (2)	
S2	0.81997 (11)	0.34262 (7)	0.04922 (6)	0.0250 (2)	
N1	0.7013 (4)	0.1337 (3)	0.5349 (2)	0.0272 (6)	
H1N1	0.721 (5)	0.173 (3)	0.577 (2)	0.044 (11)*	
H1N2	0.680 (6)	0.0521 (16)	0.568 (3)	0.053 (13)*	
N2	0.6604 (3)	0.3220 (2)	0.39282 (18)	0.0179 (5)	
H2N	0.642 (5)	0.354 (3)	0.3237 (10)	0.042 (11)*	
N3	0.7031 (3)	0.3921 (2)	0.45314 (18)	0.0171 (5)	
N4	0.7758 (4)	0.7985 (2)	0.45494 (19)	0.0208 (5)	
H4N	0.762 (5)	0.8832 (11)	0.440 (3)	0.040 (11)*	
N5	0.5325 (4)	0.4371 (3)	0.1469 (2)	0.0239 (6)	
H5N1	0.450 (4)	0.502 (2)	0.147 (3)	0.037 (10)*	
H5N2	0.487 (5)	0.365 (2)	0.154 (3)	0.037 (10)*	
N6	0.7531 (4)	0.5739 (2)	0.0624 (2)	0.0223 (5)	
H6N	0.855 (3)	0.590 (4)	0.021 (3)	0.047 (11)*	
N7	0.6520 (3)	0.6664 (2)	0.09750 (19)	0.0199 (5)	
N8	0.6103 (3)	1.0744 (2)	0.11636 (19)	0.0210 (5)	
H8N	0.640 (5)	1.1508 (18)	0.111 (3)	0.037 (10)*	
C1	0.6607 (4)	0.1932 (3)	0.4337 (2)	0.0173 (6)	
C2	0.6936 (4)	0.5146 (3)	0.4011 (2)	0.0175 (6)	
H2	0.6579	0.5459	0.3296	0.021*	
C3	0.7347 (4)	0.6064 (3)	0.4466 (2)	0.0161 (6)	
C4	0.7238 (4)	0.7375 (3)	0.3922 (2)	0.0196 (6)	
H4	0.6857	0.7794	0.3213	0.024*	
C5	0.7953 (4)	0.5851 (3)	0.5511 (2)	0.0148 (5)	
C6	0.8287 (4)	0.4758 (3)	0.6422 (2)	0.0188 (6)	
H6	0.8136	0.3924	0.6426	0.023*	
C7	0.8843 (4)	0.4918 (3)	0.7319 (2)	0.0230 (6)	
H7	0.9067	0.4184	0.7944	0.028*	
C8	0.9078 (4)	0.6146 (3)	0.7319 (2)	0.0237 (7)	
H8	0.9464	0.6227	0.7943	0.028*	
C9	0.8759 (4)	0.7244 (3)	0.6426 (2)	0.0211 (6)	
H9	0.8925	0.8074	0.6426	0.025*	
C10	0.8183 (4)	0.7082 (3)	0.5529 (2)	0.0174 (6)	
C11	0.6925 (4)	0.4572 (3)	0.0883 (2)	0.0192 (6)	
C12	0.7333 (4)	0.7692 (3)	0.0773 (2)	0.0189 (6)	
H12	0.8548	0.7738	0.0434	0.023*	
C13	0.6459 (4)	0.8771 (3)	0.1045 (2)	0.0164 (6)	
C14	0.7289 (4)	0.9880 (3)	0.0867 (2)	0.0191 (6)	
H14	0.8527	1.0017	0.0576	0.023*	
C15	0.4594 (4)	0.8983 (3)	0.1488 (2)	0.0156 (6)	
C16	0.3047 (4)	0.8256 (3)	0.1817 (2)	0.0210 (6)	
H16	0.3122	0.7412	0.1790	0.025*	
C17	0.1416 (4)	0.8801 (3)	0.2180 (2)	0.0247 (7)	
H17	0.0372	0.8312	0.2414	0.030*	
C18	0.1256 (4)	1.0053 (3)	0.2214 (2)	0.0245 (7)	
H18	0.0106	1.0403	0.2453	0.029*	
C19	0.2771 (4)	1.0783 (3)	0.1898 (2)	0.0231 (6)	
H19	0.2682	1.1629	0.1922	0.028*	
C20	0.4422 (4)	1.0232 (3)	0.1547 (2)	0.0185 (6)	

### Atomic displacement parameters (Å^2^)

**Table:**

	*U*^11^	*U*^22^	*U*^33^	*U*^12^	*U*^13^	*U*^23^
S1	0.0312 (4)	0.0162 (4)	0.0216 (4)	−0.0052 (3)	−0.0014 (3)	−0.0101 (3)
S2	0.0274 (4)	0.0203 (4)	0.0321 (4)	−0.0073 (3)	0.0061 (3)	−0.0150 (3)
N1	0.0433 (17)	0.0196 (14)	0.0201 (13)	−0.0059 (13)	−0.0052 (12)	−0.0070 (11)
N2	0.0242 (13)	0.0160 (12)	0.0171 (12)	−0.0055 (10)	−0.0017 (10)	−0.0089 (10)
N3	0.0168 (12)	0.0196 (13)	0.0199 (12)	−0.0058 (10)	0.0008 (9)	−0.0120 (10)
N4	0.0269 (13)	0.0135 (13)	0.0233 (13)	−0.0045 (11)	−0.0012 (10)	−0.0072 (10)
N5	0.0218 (13)	0.0207 (14)	0.0303 (14)	−0.0038 (11)	0.0041 (11)	−0.0108 (12)
N6	0.0231 (13)	0.0180 (13)	0.0280 (14)	−0.0031 (11)	0.0032 (11)	−0.0114 (11)
N7	0.0221 (12)	0.0150 (12)	0.0232 (12)	0.0008 (10)	−0.0018 (10)	−0.0081 (10)
N8	0.0269 (14)	0.0162 (13)	0.0244 (13)	−0.0094 (11)	0.0015 (10)	−0.0105 (10)
C1	0.0165 (13)	0.0175 (14)	0.0188 (14)	−0.0034 (11)	0.0013 (11)	−0.0074 (11)
C2	0.0157 (13)	0.0213 (15)	0.0173 (13)	−0.0058 (12)	0.0007 (10)	−0.0079 (11)
C3	0.0144 (13)	0.0178 (14)	0.0177 (13)	−0.0029 (11)	0.0015 (10)	−0.0081 (11)
C4	0.0223 (15)	0.0182 (15)	0.0188 (14)	−0.0035 (12)	−0.0005 (11)	−0.0069 (12)
C5	0.0110 (12)	0.0163 (14)	0.0194 (13)	−0.0042 (11)	0.0029 (10)	−0.0089 (11)
C6	0.0176 (14)	0.0181 (15)	0.0220 (14)	−0.0036 (12)	−0.0004 (11)	−0.0080 (12)
C7	0.0224 (15)	0.0254 (16)	0.0189 (14)	−0.0020 (13)	−0.0019 (12)	−0.0051 (12)
C8	0.0193 (14)	0.0349 (18)	0.0221 (15)	−0.0027 (13)	−0.0025 (12)	−0.0159 (13)
C9	0.0171 (14)	0.0239 (16)	0.0279 (15)	−0.0020 (12)	−0.0003 (12)	−0.0163 (13)
C10	0.0138 (13)	0.0175 (15)	0.0225 (14)	−0.0024 (11)	0.0023 (11)	−0.0096 (12)
C11	0.0210 (15)	0.0170 (15)	0.0215 (14)	−0.0031 (12)	−0.0030 (11)	−0.0083 (12)
C12	0.0204 (14)	0.0175 (15)	0.0176 (13)	−0.0009 (12)	−0.0011 (11)	−0.0053 (11)
C13	0.0206 (14)	0.0125 (14)	0.0152 (13)	−0.0041 (11)	−0.0022 (11)	−0.0025 (11)
C14	0.0199 (14)	0.0208 (15)	0.0177 (13)	−0.0047 (12)	−0.0003 (11)	−0.0072 (12)
C15	0.0175 (13)	0.0149 (14)	0.0133 (13)	−0.0013 (11)	−0.0039 (10)	−0.0030 (11)
C16	0.0237 (15)	0.0165 (15)	0.0216 (14)	−0.0060 (12)	−0.0035 (12)	−0.0032 (12)
C17	0.0172 (14)	0.0294 (17)	0.0258 (15)	−0.0066 (13)	−0.0009 (12)	−0.0065 (13)
C18	0.0201 (15)	0.0312 (18)	0.0223 (15)	0.0027 (13)	−0.0005 (12)	−0.0114 (13)
C19	0.0299 (16)	0.0206 (16)	0.0207 (14)	0.0018 (13)	−0.0057 (12)	−0.0101 (12)
C20	0.0237 (15)	0.0180 (14)	0.0161 (13)	−0.0040 (12)	−0.0050 (11)	−0.0072 (11)

### Geometric parameters (Å, °)

**Table:**

S1—C1	1.696 (3)	C4—H4	0.9500
S2—C11	1.689 (3)	C5—C6	1.399 (4)
N1—C1	1.329 (4)	C5—C10	1.415 (4)
N1—H1N1	0.87 (3)	C6—C7	1.388 (4)
N1—H1N2	0.89 (3)	C6—H6	0.9500
N2—C1	1.341 (4)	C7—C8	1.403 (4)
N2—N3	1.393 (3)	C7—H7	0.9500
N2—H2N	0.887 (10)	C8—C9	1.386 (4)
N3—C2	1.289 (4)	C8—H8	0.9500
N4—C4	1.365 (3)	C9—C10	1.395 (4)
N4—C10	1.376 (4)	C9—H9	0.9500
N4—H4N	0.886 (10)	C12—C13	1.436 (4)
N5—C11	1.335 (4)	C12—H12	0.9500
N5—H5N1	0.88 (3)	C13—C14	1.379 (4)
N5—H5N2	0.88 (3)	C13—C15	1.446 (4)
N6—C11	1.344 (4)	C14—H14	0.9500
N6—N7	1.385 (3)	C15—C16	1.409 (4)
N6—H6N	0.88 (3)	C15—C20	1.414 (4)
N7—C12	1.287 (4)	C16—C17	1.384 (4)
N8—C14	1.350 (4)	C16—H16	0.9500
N8—C20	1.384 (4)	C17—C18	1.405 (4)
N8—H8N	0.88 (3)	C17—H17	0.9500
C2—C3	1.443 (4)	C18—C19	1.391 (4)
C2—H2	0.9500	C18—H18	0.9500
C3—C4	1.376 (4)	C19—C20	1.391 (4)
C3—C5	1.443 (4)	C19—H19	0.9500
			
C1—N1—H1N1	124 (3)	C8—C7—H7	119.5
C1—N1—H1N2	120 (3)	C9—C8—C7	121.4 (3)
H1N1—N1—H1N2	114 (4)	C9—C8—H8	119.3
C1—N2—N3	121.7 (2)	C7—C8—H8	119.3
C1—N2—H2N	113 (2)	C8—C9—C10	117.4 (3)
N3—N2—H2N	125 (2)	C8—C9—H9	121.3
C2—N3—N2	113.1 (2)	C10—C9—H9	121.3
C4—N4—C10	109.1 (2)	N4—C10—C9	129.9 (3)
C4—N4—H4N	125 (2)	N4—C10—C5	108.0 (2)
C10—N4—H4N	125 (2)	C9—C10—C5	122.1 (3)
C11—N5—H5N1	121 (2)	N5—C11—N6	117.1 (3)
C11—N5—H5N2	115 (2)	N5—C11—S2	123.0 (2)
H5N1—N5—H5N2	116 (3)	N6—C11—S2	119.9 (2)
C11—N6—N7	120.0 (2)	N7—C12—C13	122.0 (3)
C11—N6—H6N	118 (3)	N7—C12—H12	119.0
N7—N6—H6N	122 (3)	C13—C12—H12	119.0
C12—N7—N6	114.4 (2)	C14—C13—C12	124.2 (3)
C14—N8—C20	109.3 (2)	C14—C13—C15	105.9 (2)
C14—N8—H8N	123 (2)	C12—C13—C15	129.9 (2)
C20—N8—H8N	127 (2)	N8—C14—C13	110.7 (3)
N1—C1—N2	117.8 (2)	N8—C14—H14	124.6
N1—C1—S1	123.6 (2)	C13—C14—H14	124.6
N2—C1—S1	118.6 (2)	C16—C15—C20	118.8 (3)
N3—C2—C3	123.1 (3)	C16—C15—C13	134.5 (3)
N3—C2—H2	118.5	C20—C15—C13	106.7 (2)
C3—C2—H2	118.5	C17—C16—C15	118.4 (3)
C4—C3—C2	123.6 (3)	C17—C16—H16	120.8
C4—C3—C5	106.6 (2)	C15—C16—H16	120.8
C2—C3—C5	129.8 (3)	C16—C17—C18	122.0 (3)
N4—C4—C3	110.1 (3)	C16—C17—H17	119.0
N4—C4—H4	125.0	C18—C17—H17	119.0
C3—C4—H4	125.0	C19—C18—C17	120.4 (3)
C6—C5—C10	119.4 (2)	C19—C18—H18	119.8
C6—C5—C3	134.3 (2)	C17—C18—H18	119.8
C10—C5—C3	106.3 (2)	C20—C19—C18	117.7 (3)
C7—C6—C5	118.6 (3)	C20—C19—H19	121.2
C7—C6—H6	120.7	C18—C19—H19	121.2
C5—C6—H6	120.7	N8—C20—C19	129.9 (3)
C6—C7—C8	121.1 (3)	N8—C20—C15	107.4 (2)
C6—C7—H7	119.5	C19—C20—C15	122.6 (3)
			
C1—N2—N3—C2	−179.2 (2)	C3—C5—C10—C9	179.8 (2)
C11—N6—N7—C12	173.1 (3)	N7—N6—C11—N5	0.9 (4)
N3—N2—C1—N1	0.7 (4)	N7—N6—C11—S2	−177.8 (2)
N3—N2—C1—S1	−178.20 (19)	N6—N7—C12—C13	177.7 (2)
N2—N3—C2—C3	−179.0 (2)	N7—C12—C13—C14	178.5 (3)
N3—C2—C3—C4	−179.5 (3)	N7—C12—C13—C15	−4.9 (5)
N3—C2—C3—C5	1.5 (5)	C20—N8—C14—C13	−0.3 (3)
C10—N4—C4—C3	−1.2 (3)	C12—C13—C14—N8	177.4 (2)
C2—C3—C4—N4	−178.3 (3)	C15—C13—C14—N8	0.1 (3)
C5—C3—C4—N4	0.9 (3)	C14—C13—C15—C16	178.4 (3)
C4—C3—C5—C6	178.6 (3)	C12—C13—C15—C16	1.3 (5)
C2—C3—C5—C6	−2.3 (5)	C14—C13—C15—C20	0.1 (3)
C4—C3—C5—C10	−0.2 (3)	C12—C13—C15—C20	−177.0 (3)
C2—C3—C5—C10	178.9 (3)	C20—C15—C16—C17	0.3 (4)
C10—C5—C6—C7	−0.2 (4)	C13—C15—C16—C17	−177.8 (3)
C3—C5—C6—C7	−178.8 (3)	C15—C16—C17—C18	1.0 (4)
C5—C6—C7—C8	−0.3 (4)	C16—C17—C18—C19	−1.4 (4)
C6—C7—C8—C9	0.3 (4)	C17—C18—C19—C20	0.4 (4)
C7—C8—C9—C10	0.4 (4)	C14—N8—C20—C19	−177.0 (3)
C4—N4—C10—C9	−179.3 (3)	C14—N8—C20—C15	0.4 (3)
C4—N4—C10—C5	1.0 (3)	C18—C19—C20—N8	178.0 (3)
C8—C9—C10—N4	179.5 (3)	C18—C19—C20—C15	0.9 (4)
C8—C9—C10—C5	−0.9 (4)	C16—C15—C20—N8	−178.9 (2)
C6—C5—C10—N4	−179.5 (2)	C13—C15—C20—N8	−0.3 (3)
C3—C5—C10—N4	−0.5 (3)	C16—C15—C20—C19	−1.3 (4)
C6—C5—C10—C9	0.8 (4)	C13—C15—C20—C19	177.4 (3)

### Hydrogen-bond geometry (Å, °)

**Table:**

*D*—H···*A*	*D*—H	H···*A*	*D*···*A*	*D*—H···*A*
N4—H4n···S1^i^	0.89 (1)	2.56 (2)	3.383 (3)	156 (3)
N8—H8n···S2^i^	0.88 (3)	2.49 (2)	3.325 (2)	157 (3)

Symmetry codes: (i) *x*, *y*+1, *z*.
